# Disentangling Component Dynamics in an All-Polymer Nanocomposite Based on Single-Chain Nanoparticles by Quasielastic Neutron Scattering

**DOI:** 10.1021/acs.macromol.1c02382

**Published:** 2022-02-28

**Authors:** Jon Maiz, Ester Verde-Sesto, Isabel Asenjo-Sanz, Lucile Mangin-Thro, Bernhard Frick, José A. Pomposo, Arantxa Arbe, Juan Colmenero

**Affiliations:** † Centro de Física de Materiales (CFM) (CSIC-UPV/EHU)-Materials Physics Center (MPC), Paseo Manuel de Lardizábal 5, 20018 Donostia-San Sebastián, Spain; ‡ IKERBASQUE-Basque Foundation for Science, Plaza Euskadi 5, 48009 Bilbao, Spain; § Institut Laue-Langevin, 71 Avenue des Martyrs, 38042 Grenoble Cedex 9, France; ∥ Departamento de Polímeros y Materiales Avanzados: Física, Química y Tecnología, Universidad del País Vasco-Euskal Herriko Unibertsitatea (UPV/EHU), 20018 Donostia-San Sebastián, Spain; ⊥ Donostia International Physics Center, Paseo Manuel de Lardizábal 4, 20018 Donostia-San Sebastián, Spain

## Abstract

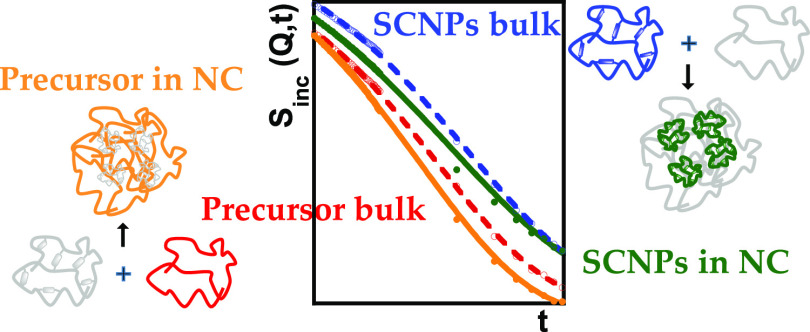

We have investigated an all-polymer nanocomposite (NC) consisting of single-chain nanoparticles (SCNPs) immersed in a matrix of linear chains of their precursors (25/75% composition in weight). The SCNPs were previously synthesized via “click” chemistry, which induces intramolecular cross-links in the individual macromolecules accompanied by a slight shift (5–8 K) of the glass transition temperature toward higher values and a broadening of the dynamic response with respect to the raw precursor material. The selective investigation of the dynamics of the NC components has been possible by using properly isotopically labeled materials and applying quasielastic neutron scattering techniques. Results have been analyzed in the momentum transfer range where the coherent scattering contribution is minimal, as determined by complementary neutron diffraction experiments with polarization analysis. We observe the development of dynamic heterogeneity in the intermediate scattering function of the NC components, which grows with increasing time. Local motions in the precursor matrix of the NC are accelerated with respect to the reference bulk behavior, while the displacements of SCNPs’ hydrogens show enhanced deviations from Gaussian and exponential behavior compared with the pure melt of SCNPs. The resulting averaged behavior in the NC coincides with that of the pure precursor, in accordance with the macroscopic observations by differential scanning calorimetry (DSC) experiments.

## Introduction

Research on polymer nanocomposites, materials composed of a polymer matrix with embedded fillers, is nowadays of industrial and academic interest.^1,2^ The growing interest in applications of such materials can be traced back to the different properties of the final nanocomposite material compared to the pure polymer matrix. In nanocomposites, several parameters can be varied and/or tuned to improve their final properties. Not only it is possible to change matrix parameters (e.g., chemistry, architecture, and molecular weight) and/or fillers’ parameters (e.g., shape, surface, and size), but also parameters related with the final mixture (e.g., composition and solvent interaction). Concerning the fillers, different classes of nanofillers have been used in polymer nanocomposites over the last years. Particularly, so-called polymer nanoparticles,^3−10^ involving polymeric materials, have attracted the interest of many research groups. This kind of nanocomposites, also called all-polymer nanocomposites, has the advantage that materials can be designed and prepared for which the size and “softness” of the dispersed components are highly tunable.^11^

A new family of polymer nanoparticles called single-chain nanoparticles (SCNPs) has emerged over the last years. SCNPs are unimolecular nano-objects obtained by intramolecular cross-linking of individual macromolecular chains (functionalized linear polymers called “precursors”).^12−15^ Several studies have been published where these SCNPs were blended with a linear polymer matrix to give all-polymer nanocomposites, and different aspects of the resulting mixtures have been investigated. For example, direct experimental observation of the miscibility was reported for SCNPs based on polystyrene (PS) blended with poly(vinyl methyl ether) (PVME) linear polymer chains.^16^ Interestingly, it was found a very different calorimetric and dielectric behavior of PS/PVME mixtures depending on whether the PS-component consisted of linear chains or of SCNPs.^17,18^ In addition, the structural properties as well as the thermal behavior, the segmental and chain dynamics of systems based on poly(methyl methacrylate) (PMMA) SCNPs blended with linear poly(ethylene oxide) (PEO), were also studied during the last years.^19−21^ Confinement effects on the PEO chains were reported. It is worth noting that both families of mixtures, composed by PMMA/PEO and consisting of PVME/PS, present an inherent strong dynamic asymmetry (difference in the values of the glass transition temperature (*T*_g_) of the two pure components), that could, at least partially, be at the origin of the peculiar phenomenology found in these materials. This dynamic asymmetry was absent in the components of the nanocomposites based on PS investigated in the literature.^22^ There, linear PS chains were mixed with PS–SCNPs. In that work, the mechanical and thermal behavior of the mixtures were investigated, reporting a reduction of the viscosity and the *T*_g_ with respect to the linear chains, even if the *T*_g_-values of the two pure components were equal. Thus, even in systems where ingredients introducing additional complexity to the problem are minimized, an intriguing phenomenology is found. In particular, the glass transition phenomenon seems to be affected by the SCNP nature of one of the components. The glass transition is closely related to the dynamics of the segmental or α-relaxation; information about the atomic motions involved in this dynamical process is thus of utmost importance to understand the observed behavior in these mixtures. In particular, we need to determine the mutual influence of the components in their atomic motions in the α-relaxation regime. This question has been extensively investigated in the case of blends of linear chains.^23,24^ But little is known about what happens when one of the components in the mixture consists of SCNPs.^19−21^ Molecular dynamics (MD) simulations can address this question, as it was the case of the works reported in the literature for PS-based nanocomposites.^25,26^ However, from an experimental point of view, this is a rather complicated problem, since component-selective techniques are required. The quasielastic neutron scattering (QENS) technique applied to isotopically labeled samples is the right tool to shed light on this problem because it directly accesses atomic motions of selected components in the system at the molecular level. In this context, the aim of this work is to investigate the phenomenon of the glass transition and the component segmental dynamics associated with it in a nanocomposite consisting of SCNPs embedded in a linear-chain matrix of their precursors, combining calorimetry and QENS techniques.

QENS techniques are based on the fact that the scattering of a neutron by a nucleus can alter its momentum and energy.^27,28^ The momentum transfer dependence of the scattered intensity provides space resolution, and the energy dependence, time resolution, both at the microscopic level. The double differential scattering cross section ∂^2^σ/∂Ω∂*E* determined in a QENS experiment is the number of neutrons scattered into a solid angle between Ω and Ω + ∂Ω with an energy change Δ*E* = ℏω.^29^ Elastic scattering occurs when there is no energy exchange (within the instrumental resolution) between the atoms and the neutrons. In inelastic scattering, neutrons loose or gain energy related to excitations in the sample. Quasielastic scattering^30^ gives rise to broadening around elastic lines reflecting stochastic and diffusive motions, relaxations, etc. in the sample.^31^

The double differential scattering cross section contains coherent and incoherent contributions. Coherent scattering gives information related to collective properties, while incoherent scattering is associated with self-motions. The respective weight of the coherent and incoherent contributions to the total scattering cross section is determined by the coherent and incoherent scattering lengths of the nuclei in the sample.^31,32^ The scattering length *b* characterizes the strength of the neutron–nucleus interaction; it depends on the relative orientation of the neutron–nuclear spin pairs and varies from one isotope to another. In particular, hydrogen presents a huge incoherent scattering cross section σ (σ_inc_^H^ ≈ 80 barn) compared to the coherent (σ_coh_^D^ ≈ 5.6 barn) and incoherent (σ_inc_^D^ ≈ 2.0 barn) cross sections of deuterons (the scattering cross section σ is defined as σ = 4π*b*^2^). Therefore, deuteration of a given moiety or component in a sample drastically reduces its cross section for neutrons, and the intensity scattered by the sample is generally dominated by the incoherent contribution from the remaining hydrogens in the system (see below). Due to this capability to selectively investigate components in a complex material, neutron scattering techniques are extremely useful to study the dynamics in nanocomposite materials.

**Scheme 1:**
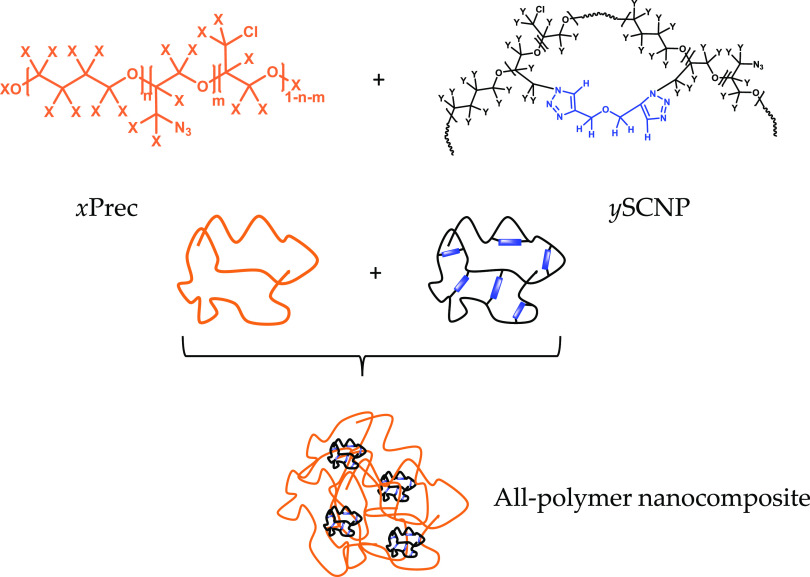
Schematic Illustration of All-Polymer Nanocomposite Composition The samples are prepared by mixing the PTHF precursor with the PTHF-based SCNPs. The formed nanocomposite is composed by *x*Prec/*y*SCNPs samples, on selectively labeled (*x*,*y*)ϵ(*h*,*d*) samples. Two kinds of nanocomposites are prepared in the present work, the first one 75hPrec/25dSCNPs sample and the second sample was the inversely labeled one 75dPrec/25hSCNPs. All of the synthesis methods and procedures are described in detail in refs (33) and (34).

In the present work, we study a mixture consisting of 75 wt % poly(tetrahydrofuran) (PTHF)-based linear precursor chains and 25 wt % of PTHF SCNPs. Two different samples are investigated, where the protonated (*h*) and deuterated (*d*) moieties are interchanged on selectively labeled (*h*/*d*) samples, as shown in Scheme 1. In the sample where the SCNPs are protonated and the precursors are deuterated, the scattered intensity in the accessed dynamic window is dominated by the self-atomic motions of the hydrogens in the SCNPs. In the inversely labeled sample, we follow the precursors′ hydrogen motions. Thus, with our QENS experiments, we can discern how the dynamics of both components are mutually affected. To cover a wide dynamic range, we have combined two kinds of QENS spectrometers, a backscattering (BS) and a time-of-flight (ToF) instrument. The *Q*-range accessed (approx. 0.2 ≤ *Q* ≤ 2 Å^–1^, *Q*: modulus of the scattering vector; ℏ*Q*: momentum transfer) corresponds to relatively local length scales of observation (these are inversely proportional to the *Q*-scales explored). Our QENS experiments are complemented by diffraction experiments with polarization analysis, to determine the ratio between coherent and incoherent differential cross sections in the samples. This allows to discern in which regions of *Q*, Bragg-peaks or concentration fluctuations are present and we have to be cautious with the coherent contribution to the scattered intensity. The raw materials were previously studied and published by the authors.^33,34^

## Experimental Section

### Materials

Briefly, all chemical reagents and solvents were obtained from Sigma-Aldrich (Munich, Germany), Scharlab (Barcelona, Spain), and Eurisotop (Saint-Aubin, France). The materials used and the purification methods applied are published in previous works.^33−35^

### Synthesis Methods

Both protonated and deuterated synthetic routes employed to prepare tetrahydrofuran (THF) and epichlorohydrin (ECH) (P(THF-*co*-ECH)) copolymers have been reported in our previous works.^33,34^ The synthesis of single-chain nanoparticles (SCNPs) was carried out via copper(I)-catalyzed azide alkyne cycloaddition (CuAAc) “click” reaction. All of the procedures are also reported in refs (33) and (34). Molecular weight and polydispersity were 22 kg/mol and 1.24 for the protonated polymers and 36.5 kg/mol and 1.20 for the deuterated polymers, respectively.

### Sample Preparation

Two different samples of nanocomposites containing 75 wt % of the precursor and 25 wt % of SCNPs were prepared. Blends were prepared by mixing the appropriate precursor with the SCNP sample to prepare either the hPrec/dSCNP or dPrec/hSCNP mixture, where *h* represents protonated samples and *d* symbolizes deuterated ones. First, the precursor and the SCNP mixtures were stirred until the total dissolution in CH_2_Cl_2_ for 24 h at room temperature. Then, each mixture was drop-casted onto the aluminum flat holders used for neutron scattering experiments. Subsequently, the solvent was slowly evaporated in a fume hood, and finally, the samples were well-dried in an oven at 343 K for 24 h under vacuum conditions. The appearance of the samples was as that of a viscous fluid.

### Thermal Analysis

Glass transition temperatures (*T*_g_) were studied by differential scanning calorimetry (DSC). DSC experiments were carried out using a TA instrument Q2000 under ultrapure nitrogen flow. For each experiment, around 5 mg of the polymer mass was used, and from the onset of the heat flow jump, the *T*_g_ values were extracted. The following protocol was applied in all of the samples studied. First, the samples were heated up until 350 K and kept there for 3 min to erase any previous thermal history. Then, they were cooled down until 170 K at 20 K/min, and afterward, from this temperature, they were again heated at 20 K/min until 350 K.

### Structural Analysis

Neutron diffraction with polarization analysis was carried out in a neutron scattering experiment for the separation of coherent and incoherent differential cross sections.^19,36^ The probability of the change of the spin direction of the neutron for completely unpolarized nuclei is 2/3 for scattering with flip and 1/3 for scattering without flip. The fact that the scattered intensity with spin flip results only from the incoherent part is used to separate coherent and incoherent scattering. D7 experiments at Institute Laue Langevin (ILL) in Grenoble, France,^37^ were carried out where incident neutron wavelength was set to λ = 4.89 Å to cover a *Q*-range from 0.15 to 2.5 Å^–1^. A Vanadium sheet was used to calibrate the detector efficiency, and for the background scattering, a combination of an empty cell and a Cadmium sheet was used. In addition, a quartz plate for the polarization correction was used. The samples were studied at 300 K and flat aluminum cells were used as sample holders.

### Quasielastic Neutron Scattering (QENS) Analysis

To access a wide QENS dynamic range, two different spectrometers were combined. In this study, the backscattering (BS) IN16B spectrometer and the time-of-flight (ToF) IN5 instrument, both located at ILL, were used.^37^ With them, the *Q*-range covered was from 0.19 to 1.90 Å^–1^. The temperatures investigated were 285, 320, and 360 K. The sample thicknesses used in the experiments were calculated to reach around 90% of transmission, and flat aluminum cells were used as sample holders. The incident wavelength λ was 6.271 Å for the IN16B spectrometer, while λ = 5 Å was used for the IN5 instrument. To analyze the quasielastic spectra, the data were Fourier-transformed to the time domain, and then, a deconvolution was done from the instrumental resolution to obtain the intermediate scattering function in the time domain *S*(*Q*,*t*). Due to limited beam time, a low-temperature measurement, ideal for the accurate normalization of the deconvoluted results, could not be performed in all samples investigated. Spectra obtained on a Vanadium sample were used to perform the deconvolution in the case of IN16B results. Mismatches when putting them together with the time-of-flight data were corrected by applying scaling factors to the backscattering data.

## Results

**Figure 1:**
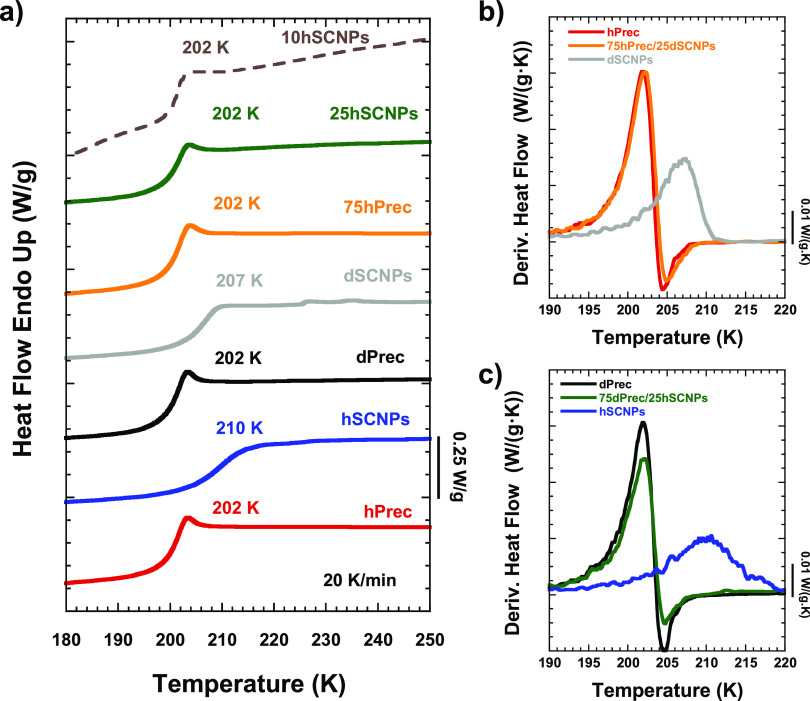
DSC heating scans at 20 K/min after the previous cooling process of previously reported protonated and deuterated precursors and SCNPs, and the nanocomposites here investigated. Temperature evolution of the derivative of the heat flow of the (b) 75hPrec/25dSCNPs sample and its pure components and (c) 75dPrec/25hSCNPs sample and its pure components. Results corresponding to a sample with 10% hSCNPs in 90% dPrec are also shown in (a), dashed line.

We first present the calorimetric results. In Figure 1a, the DSC heating scans and *T*_g_ values of the nanocomposites as well as of their pure components (protonated (*h*) and deuterated (*d*) precursors and SCNPs) are shown. Both *h*- and *d*- precursor *T*_g_ values were 202 K, while in the SCNPs, they increase up to 210 and 207 K, respectively. Independently of the isotopic label, when the composition of the nanocomposite is 75 wt % precursor and 25 wt % SCNPs, the *T*_g_ value is equal to the initial precursor value (202 K). The similarity applies not only to the average value of the glass transition but also to its width. This can be better appreciated in the right panels of Figure 1, where the derivative of the heat flow is compared for the mixture and its pure components. Figure 1b shows these functions for the 75hPrec/25dSCNPs sample and Figure 1c for the inverse labeling.

**Figure 2:**
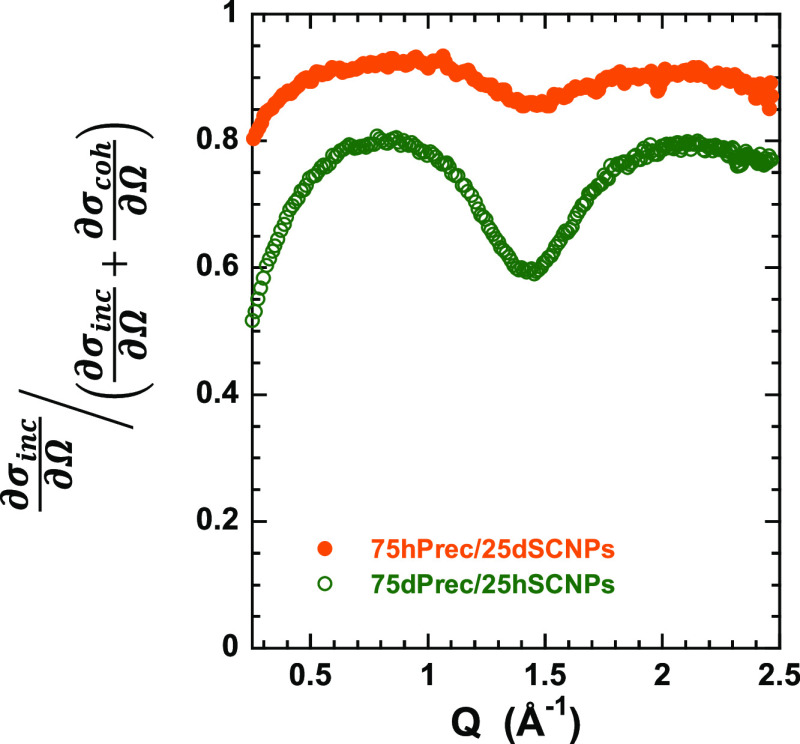
Ratio between incoherent and total differential cross sections obtained by D7 at 300 K on samples with protonated and deuterated nanocomposites.

Moving to the neutron scattering study, we consider first the information provided by the diffraction experiments with polarization analysis that permits the separation of coherent and incoherent contributions to the total intensity. Figure 2 shows the relative contribution of incoherent scattering to the total differential cross section for the two samples obtained by the procedure described in the Experimental Section. The results show that the incoherent contribution is dominant in all of the *Q*-range investigated. The sample with dPrec shows a higher amount of coherent scattering but always below 50%. The modulation of the curves shown in Figure 2 is due to this coherent scattering. Since the incoherent differential cross section is *Q*-independent, the results above *Q* ≈ 0.5 Å^–1^ reflect the inverse of the structure factor (coherent scattering). This shows a main peak in the *Q*-range between 1 and 2 Å^–1^ centered around 1.4 Å^–1^ for both samples. This peak is well-known from earlier X-ray experiments; it is commonly named “amorphous halo” and it is associated with the intermediate chain correlations.^33,34^ The short-range order, thus, seems to be not modified by the composition. The decrease of the relative incoherent contribution at low-*Q*-values (*Q* < 0.5 Å^–1^) is due to the coherent small-angle scattering arising from the concentration fluctuations in the mixture. We note that this low-*Q* coherent contribution is generally present in mixtures of isotopically labeled samples. From the D7 results, we can thus conclude that in the *Q*-range explored with the current QENS experiments, we are mainly sensitive to incoherent scattering of the protons in the hydrogenated component of the mixture, though the results below approx. 0.5 Å^–1^ and in the range 1.2 ≤ *Q* ≤ 1.7 Å^–1^ are contaminated by coherent contributions. The QENS results are presented in the following.

**Figure 3:**
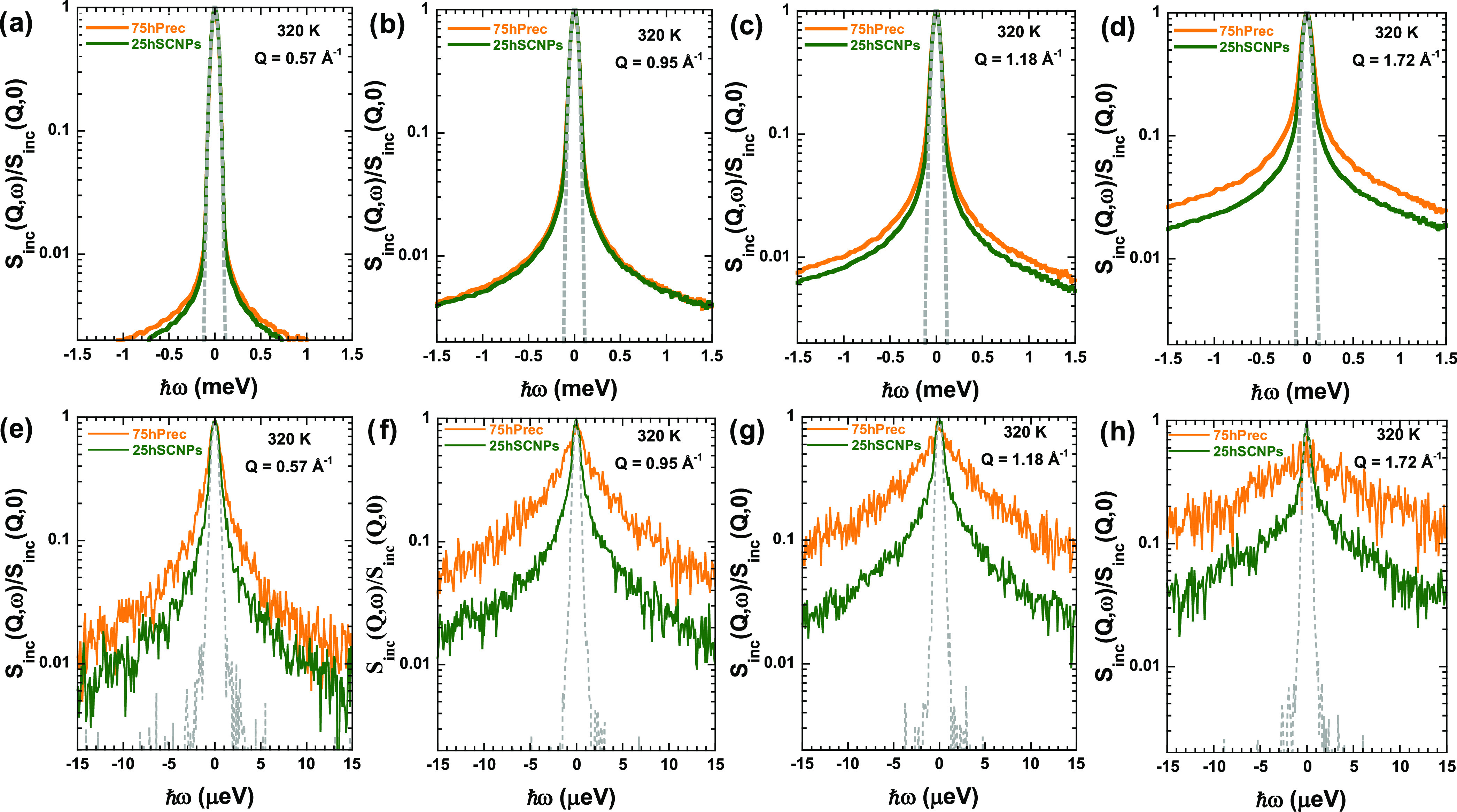
Normalized IN5 (a–d) and IN16B (e–h) spectra obtained at 320 K and the different *Q*-values indicated for different nanocomposites 75hPrec/25dSCNPs (orange line) and 75dPrec/25hSCNPs (green line). The dotted line shows the instrumental resolution function.

In a QENS experiment, the occurrence of dynamic processes with characteristic times within the experimental window of the spectrometer is observed as a broadening around the elastic line. The elastic line manifests when the energy exchanged between the sample and the neutrons is zero or smaller than the instrumental energy resolution. The resolution function *R*(*Q*,ω) is determined from the scattering of a sample where all of the dynamical processes are frozen with respect to the accessed dynamic window. As we have shown by the diffraction experiments with polarization analysis, the measured signals on our samples are dominated by the incoherent contribution. The incoherent scattering is directly related to the incoherent scattering function *S*_inc_(*Q*,ω) of the hydrogens, revealing correlations between the position of a given proton at different times in one of the nanocomposite components. Figure 3 presents representative QENS spectra. To compare directly data obtained from different samples, the curves are normalized to their value at ℏω = 0. Results at *T* = 320 K have been chosen for four representative *Q*-values in the range where the coherent contribution is minimal. At these *Q*-values, the incoherent signal amounts to 70–80% of the total intensity in the 75dPrec/25hSCNPs sample and to about 90% in the 75hPrec/25dSCNPs sample, as can be seen in Figure 2.

The width of the quasielastic spectrum is related to the inverse of the characteristic time of motion probed by the instrument. At all of the temperatures investigated and in the *Q*-range explored, we observe quasielastic broadening. For a given temperature, as can be observed in Figure 3 for the case of 320 K, this broadening becomes more pronounced with the increasing *Q*-value, which suggests the diffusive-like behavior of the protons. Moreover, we can observe a clear difference between both samples, 75hPrec/25dSCNPs and 75dPrec/25hSCNPs. In general, the spectra corresponding to the 75dPrec/25hSCNPs sample are narrower for all *Q*-values, suggesting a slower dynamics of the SCNPs with respect to the linear precursor component in both dynamic windows studied.

**Figure 4:**
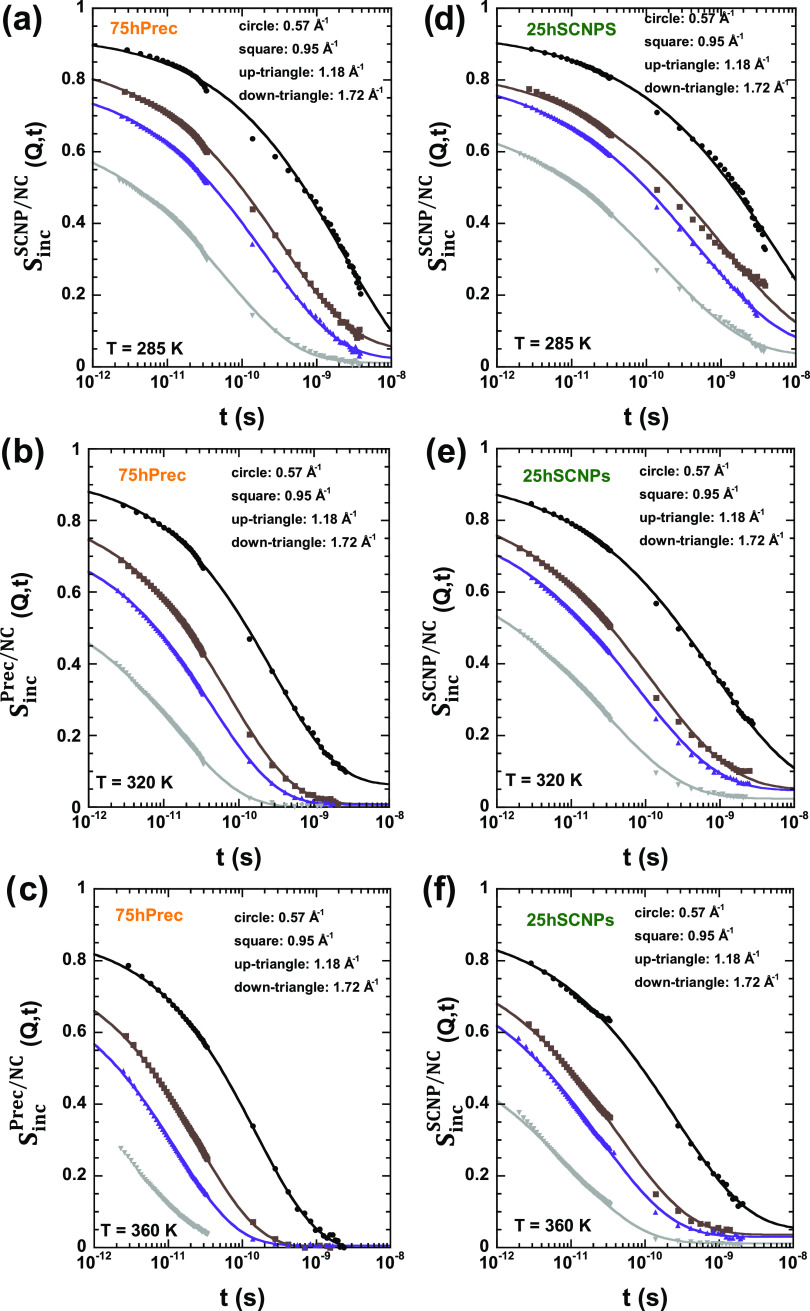
Fourier-transformed and deconvoluted QENS spectra obtained from IN5 (sets of data for times shorter than 10^–10^ s) and IN16B (sets of data for times longer than 10^–10^ s) on the 75hPrec/25dSCNPs (a–c) and 75dPrec/25hSCNPs (d–f) samples at the three different temperatures investigated: 285 K (a, d), 320 K (b, e), and 360 K (c, f) and at four different *Q*-values: 0.57 Å^–1^ (circle), 0.95 Å^–1^ (square), 1.18 Å^–1^ (up-pointing triangle), and 1.72 Å^–1^ (down-pointing triangle). Solid lines are Kohlrausch–Williams–Watts (KWW) fits with the β-values shown in Table 1 to the results above 2 ps.

**Figure 5:**
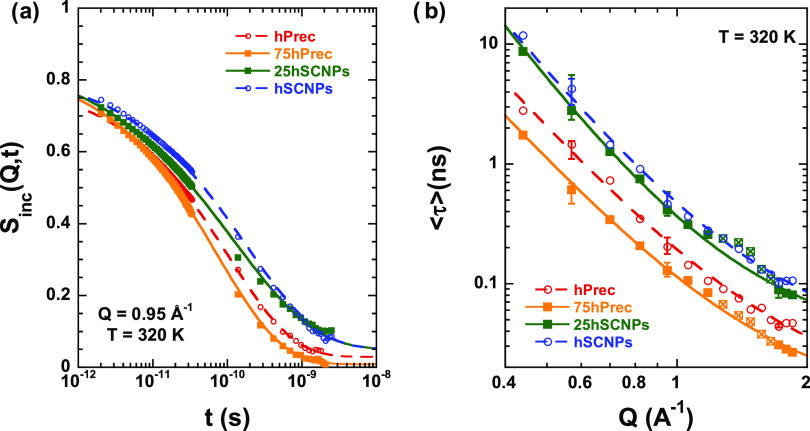
(a) Fourier-transformed and deconvoluted QENS spectra of the precursor and SCNPs (empty circles) and nanocomposites (full squares) at 320 K and *Q* = 0.95 Å^–1^. Dashed (precursor and SCNPs) and solid (nanocomposites) lines are KWW fits with the β-values shown in Table 1 to the results above 2 ps. (b) Scattering vector dependence of the average characteristic time ⟨τ⟩ = τ_s_Γ(1/β)/β for H-self-motions obtained for the precursor and SCNPs (empty circles) and nanocomposites (squares) at 320 K. Lines are fits of eq 2. Results of nanocomposites in the range 1.2 ≤ *Q* ≤ 1.65 Å^–1^ (represented by crossed squares) have been ignored for the fit of the AJD model. Estimated error bars are included for the nanocomposite results at *Q* = 0.57, 0.95, and 1.72 Å^–1^.

In this kind of experiment carried out in the frequency domain, the instrumental resolution affects the results through convolution. Consequently, the quantitative analysis of the quasielastic spectra was based on Fourier transforming the data to the time domain and deconvoluting them from the instrumental resolution effects by division. In this way, we obtained the intermediate incoherent scattering function in the time domain *S*_inc_(*Q*,*t*). This procedure has also the advantage of allowing the direct combination of results from different spectrometers. In Figure 4, the results of this procedure are shown for spectra corresponding to the same representative *Q*-values as chosen for Figure 3. From Figure 4a–c, the curves correspond to the 75hPrec/25dSCNPs sample at fixed *T*-values of 285 K (Figure 4a), 320 K (Figure 4b), and 360 K (Figure 4c). From Figure 4d–f, the results are from the 75dPrec/25hSCNPs sample at the same fixed *T*-values of 285 K (Figure 4d), 320 K (Figure 4e), and 360 K (Figure 4f). The results clearly show that the characteristic time for hydrogen motions (which, as a first approximation, can be defined as the time where *S*_inc_(*Q*,*t*) decays to 1/e) becomes shorter with increasing *T* for a given *Q*-value, and with the increasing *Q*-value at a given temperature, as we had previously deduced from the direct inspection of the spectra in the frequency domain. Also, we can appreciate that the curves show a more stretched functional form than a single exponential function. On the other hand, the intermediate incoherent scattering functions obtained for the two nanocomposite samples are compared in Figure 5a with both pure precursor and SCNP samples^33^ at the same conditions (a *Q*-value of 0.95 Å^–1^ and a fixed *T*-value of 320 K). We can see that in the pure melts the dynamics of the SCNPs is slower than that of the precursor; in the mixture, this difference appears not only to be present but even amplified at long times.

Deviations from exponential behavior are usually accounted for by Kohlrausch–Williams–Watts (KWW) or stretched exponential functions *S*_KWW_(*Q*,*t*)
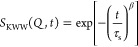
1where β is the stretching exponent characterizing the deviations from a single exponential and τ_s_ is the characteristic time that depends on both *Q* and temperature. The experimentally obtained intermediate incoherent scattering function can be well described above ≈2 ps in terms of these functions
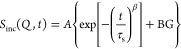
2Here, prefactor A accounts for the decay of the correlation function at shorter times due to vibrational and other fast contributions. A small elastic contribution (BG) was also allowed in the fits, which had a value of ≈0.02 and <0.05 in all cases. This component may account for an inaccurate subtraction of the background in the experiment, though it could also result from the presence of some immobile protons—within the experimental window—in the case of pronounced heterogeneous behavior. Figures 4 and 5a shows that this function works well for both nanocomposites as well as for the neat polymers^33^ in the studied *Q*-range.

**Figure 6:**
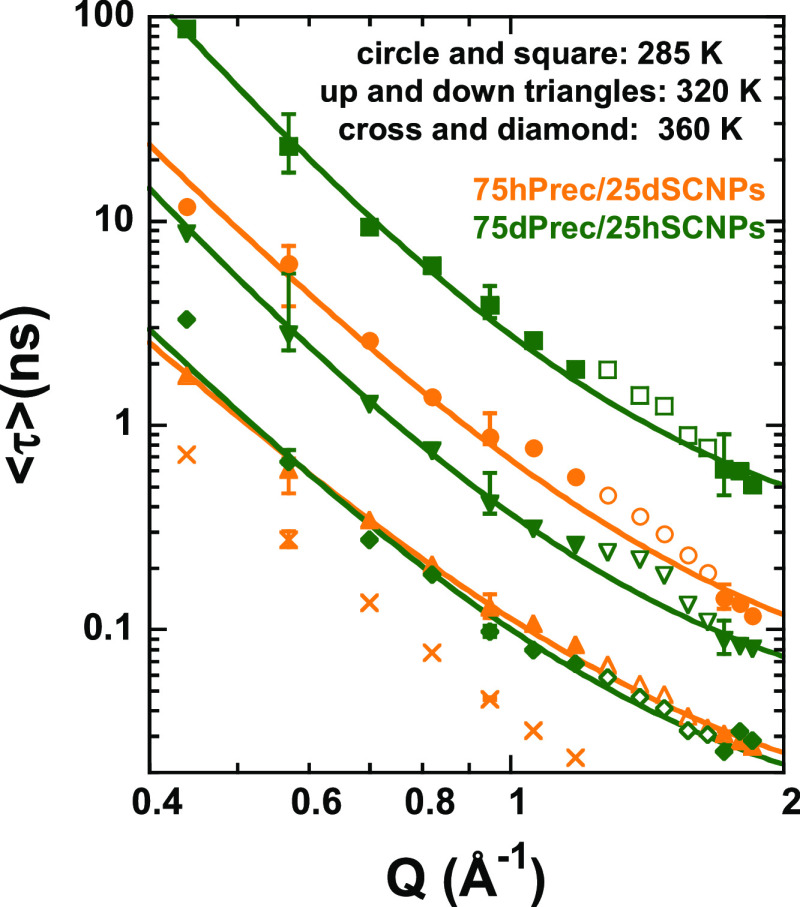
Scattering vector dependence of the average characteristic time ⟨τ⟩ = τ_s_Γ(1/β)/β for H-self-motions obtained for 75hPrec/25dSCNPs (orange symbols) and 75dPrec/25hSCNPs (green symbols) at 285 K (circle and square), 320 K (up-triangle and down-triangle), and 360 K (cross and diamond) (fits shown in Figure 4). Lines are fits of eq 2 to the 75hPrec/25dSCNPs (orange lines) and 75dPrec/25hSCNPs (green lines), using the β-values listed in Table 1 for each case. Results of nanocomposites in the range 1.2 ≤ *Q* ≤ 1.65 Å^–1^ (represented by empty symbols) have been ignored for the fit of the AJD model. Error bars have been included at *Q* = 0.57, 0.95, and 1.72 Å^–1^.

From these fits, we obtained the values of the amplitude *A*, the stretching exponent β, and the characteristic times τ_s_ as functions of *Q* and *T*. For a given temperature, the β values were scattered around the average values listed in Table 1. We then fixed the value of β to the average value at each temperature and fitted again the intermediate scattering functions, determining again the amplitude *A* and the values of the characteristic times τ_s_. The amplitude *A* follows well a Debye–Waller factor-like expression *A* ∼ exp[−<*u*^2^>*Q*^2^/3] with the values of the mean squared displacement <*u*^2^> given in Table 1. From the characteristic times τ_s_ and the β-value used, the average characteristic times were calculated. In the case of a KWW function, as expressed in eq 2, the average characteristic time is given by ⟨τ⟩ = τ_s_Γ(1/β)/β. This time is affected by the spectral shape and therefore allows comparison of results corresponding to functions with different values of the nonexponential parameter. The results on ⟨τ⟩ are collected in Figures 5b and 6. It has been reported that in the *Q*-range below approx. 1 Å^–1^ the characteristic time for H-self-motions in the α-relaxation regime of glass-forming systems^33,38−41^ follows a power law <τ> ∝ *Q*^–2/β^ as dictated by the Gaussian prediction. This can be attributed to a subdiffusive-like process. At high Qs, deviations from this Gaussian behavior manifest. To account for these experimental observations (also reported from MD simulations), the existence of an underlying distribution of jumps, giving rise to the subdiffusive regime at long times, was proposed. We remind that in jump diffusion models finite jump lengths tend to cause a bending of the dispersion for the diffusive relaxation times away from the *Q*^–2^ law, the latter being valid for simple diffusion at low-*Q*. The jump diffusion model^42−44^ considers that an atom remains in a given site for a time τ_o_, where it vibrates around a center of equilibrium. After τ_o_, it moves rapidly to a new position separated by the vector *l⃗* from its original site. It can be assumed that in a liquid or disordered system, jumps take place randomly oriented, with moduli distributed according to the function
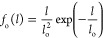
3where *l*_0_ is the preferred jump length. A generalization of these models to the case of subdiffusive behavior was proposed by the anomalous jump diffusion (AJD) model^38,39^ that introduces stretching in the time-dependent part of the intermediate scattering function. As a result, the KWW function (eq 1) representing this dynamical process in the intermediate scattering function (eq 2) has a characteristic time given by
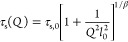
4In this way, in the limit *Ql*_o_ → 0, the Gaussian approximation is conserved, but now a sublinear increase of the mean squared displacement is obtained for low-*Q*-values; in that regime, the *Q*-dependence of τ_s_ is τ_s_ (*Q*)∝ *Q*^–2/β^. Conversely, the *Q* → ∞ limit of τ_s_ is just τ_s,o_. The value of *l*_o_ determines the *Q*-region where deviations from Gaussian behavior start (the larger are the jumps in average, the smaller is this *Q*-value).

**Table 1:** Values of the Parameters Involved in the Anomalous Jump Diffusion (AJD) Fitting the Homopolymers and Nanocomposite Mixture Results Described with KWW Functions

sample	*T* (K)	<*u*^2^> (Å^2^)	β	τ_s,0_ (ps)	*l*_0_ (Å)
	285	0.44 ± 0.02	0.44 ± 0.025	37 ± 8	0.68 ± 0.06
hPrec	320	0.55 ± 0.02	0.50 ± 0.042	5.8 ± 0.7	0.57 ± 0.04
	360	0.73 ± 0.02	0.53 ± 0.035	3.1 ± 0.3	0.69 ± 0.02
	285	0.41 ± 0.02	0.40 ± 0.037	87 ± 12	0.73 ± 0.03
hSCNPs	320	0.51 ± 0.02	0.45 ± 0.028	11.6 ± 1.2	0.63 ± 0.02
	360	0.73 ± 0.02	0.49 ± 0.030	3.6 ± 0.4	0.64 ± 0.03
	285	0.36 ± 0.04	0.44 ± 0.042	15 ± 4	0.63 ± 0.05
75hPrec/25dSCNPs	320	0.54 ± 0.04	0.50 ± 0.053	4.8 ± 0.4	0.64 ± 0.02
	360	0.57 ± 0.04	0.53 ± 0.030		
	285	0.30 ± 0.04	0.38 ± 0.035	51 ± 9	0.76 ± 0.04
75dPrec/25hSCNPs	320	0.37 ± 0.04	0.40 ± 0.060	8.9 ± 0.6	0.76 ± 0.02
	360	0.59 ± 0.04	0.44 ± 0.020	3.5 ± 1	0.73 ± 0.06

This model was applied to the results on the pure components and the two nanocomposites investigated (see descriptions in Figures 5b and 6). To avoid the influence of coherent scattering, in the fit of the results on the nanocomposites, we did not consider the data corresponding to the region 1.2 ≤ *Q* ≤ 1.65 Å^–1^, where this contribution is not negligible (see Figure 2); as can be appreciated in Figures 5b and 6, the times in this *Q*-regime show a modulation that prevents for an accurate determination of the values of the fit parameters. This model describes well the results on the pure melts and the nanocomposites (see Figures 5b and 6). The values obtained for the parameters involved in the model (*l*_0_ and τ_s,0_) are listed in Table 1. For 360 K, at high Q, the decay of the intermediate scattering function of the 75hPrec/25dSCNPs takes place with very short characteristic times, hardly distinguishable from the first fast decay below 2 ps; therefore, we did not consider the results above 1.2 Å^–1^, and consequently, we could not apply the AJD for these conditions.

## Discussion

In the pure melts, intermolecular cross-links produced by the “click” chemistry here employed induce a clear broadening of the glass transition phenomenon as monitored by DSC, with a shift of the average glass transition temperature toward higher values. These “macroscopic” effects can be appreciated in Figure 1. Broadening and slowing down effects are also manifested in the local atomic dynamics as directly observed by QENS at temperatures well above the respective *T*_g_’s of the neat samples. This can be seen in Figure 5 and deduced from the parameters describing the stretching and *Q*-dependence of the characteristic times listed in Table 1. Such findings point to more heterogeneous dynamics in the SCNPs with respect to the linear precursor counterparts, characterized microscopically by longer residence times and larger average jump lengths underlying segmental relaxation.^33,34^ In agreement with these results, recent MD simulations on melts of PS-based SCNPs have found dynamical heterogeneity and an increase in *T*_g_ of the SCNPs with respect to the linear counterparts.^45^ These effects are amplified with the increasing cross-linking degree.^45^

Thus, both, DSC and QENS show that internal cross-links induce some dynamic asymmetry in the pure components of our nanocomposites. In mixtures where the components have a marked dynamic asymmetry, as PMMA and PEO, two glass transitions associated with each of the components are found by DSC.^46^ This observation has been reported for both, blends of linear polymers^47^ as well as in all-polymer nanocomposites made of linear PEO chains as the matrix and PMMA-SCNPs,^19^ for different compositions including that here considered (75% linear matrix/25% SCNPs). However, the presence of two glass transitions is not apparent in the present mixtures. We find that the glass transition temperature of the nanocomposite basically coincides with that of the precursor (the majority component in the nanocomposite). Both, the average glass transition value and the broadening of the DSC trace are almost indistinguishable from those in the pure precursor melt (see Figure 1). Looking in detail at the derivative of the heat capacity (Figure 1b,c), a very little shift of the position of the maximum and a slightly smoother behavior of the curve could be inferred in the mixtures. However, clear effects on the glass transition of the matrix when SCNPs are added in this concentration and possible contributions of a different vitrification from the SCNPs, if any, cannot be deduced from our DSC results. We note that the macroscopic dynamic asymmetry in our samples, difference in glass transition temperatures of the homopolymers, is only about 5–8 K, i.e., much weaker than in the above-mentioned PMMA-SCNPs/PEO nanocomposites for which the difference was in the order of 50 K.^19^ The induced effects might therefore be too subtle to be resolved by standard DSC experiments.

On the contrary, the space/time insight of QENS at the molecular level does allow resolving the mutual impact of the components in the mixture. As clearly shown directly by the QENS spectra in the frequency domain, and, even more clearly, in the deconvoluted Fourier-transformed intermediate scattering functions, a dynamic heterogeneity is patent in the nanocomposites. This heterogeneity mainly arises at times longer than some picoseconds, since the values of the mean squared displacement in the fast dynamics regime <*u*^2^> are not very different for both components (see Table 1). At times longer than some picoseconds, the hydrogens in the SCNPs move in a slower fashion than the hydrogens in the precursor (see Figures 3–6). Also, the more pronounced stretching of the intermediate scattering function of the SCNPs (reflected by the β value, see Table 1) suggests significantly broader distributions of local mobilities than in the precursor component. In addition, their displacements show larger deviations from Gaussian behavior (let us remind that these deviations are quantified by the finite jump length invoked in the AJD model, *l*_0_). In terms of the AJD model, we observe clearly longer residence times and larger jump lengths for the SCNPs than for the precursor (see Table 1). We note that in the mixture, the differences are even stronger than in the separate components. This leads us to discuss the impact of each of the components on the other one, i.e., the differences induced with respect to the pure melt behavior.

The influence of the presence of the SCNPs on the dynamics of the precursor chains mainly consists of a decrease of the residence time τ_s,0_. Both the shape parameter β and the jump length *l*_0_ are not appreciably changed, within the uncertainties, with respect to those in the pure precursor (see Table 1). Interestingly enough, the effect is counterintuitive, introducing slower entities leads to speeding up the local motions in the linear chains. MD simulations on analogous nanocomposites as those here investigated, but based on PS, also reported that the segmental relaxations of the melt chains are accelerated.^25^ This effect was attributed to the thermal deformations of the loose SCNP surface structures, based on the comparison between relaxations of segments around a soft SCNP and a rigid body SCNP with the internal degrees of freedom artificially frozen. We also recall that it has been reported that under confinement, liquids and polymers can show this kind of effect.^48,49^ In those works, hard confinement was induced by a rigid surrounding environment.

**Figure 7:**
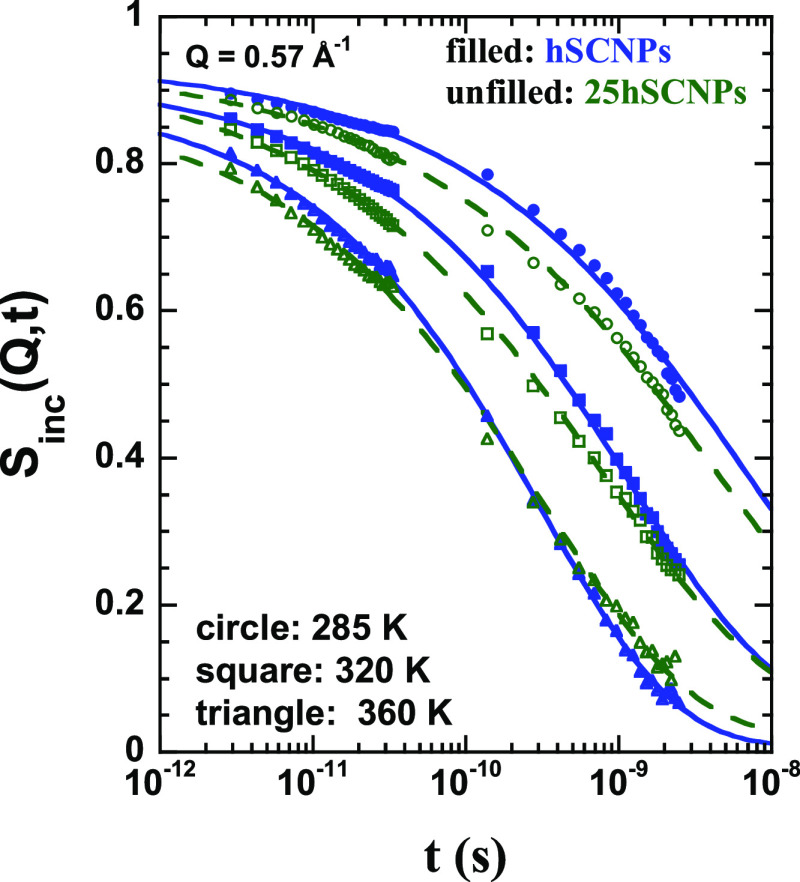
Intermediate scattering function obtained for *Q* = 0.57 Å^–1^ on the melt of SCNPs (filled symbols) and on the nanocomposite with the deuterated matrix (empty symbols), dominated by the self-motions of the hydrogens of the SCNPs in both environments. Different symbols correspond to the different temperatures indicated. Lines are fits of eq 2 with the β-values in Table 1.

Precursors modify the dynamics of SCNPs in a very different way. First of all, the intermediate scattering function of hydrogens in the SCNPs in the nanocomposite becomes even more stretched than in bulk, reflecting much more heterogeneous dynamics. This effect is clearly shown in Figure 7, where the results on the nanoparticles within the bulk and the nanocomposite samples are directly compared for *Q* = 0.57 Å^–1^ and the three temperatures investigated. We note that the observation of a mutual influence of the two components in the dynamics, leading to different behavior from the respective bulk reference systems, can be considered as proof of miscibility at the molecular level. On the other hand, while now the residence time remains practically unaffected, within the uncertainties, the jump length shows an increase with respect to the bulk. This is a signature of stronger deviations from Gaussian behavior in the atomic displacements. In the comparison of Figure 5a, we can see that the main difference between the hydrogen motions of the SCNPs in the nanocomposite and in bulk is found at short timescales. At such short times, the SCNP behavior seems to be strongly modified by the presence of the faster precursor; apparently, SCNPs follow the faster majority component at the beginning of their relaxation. However, the final decay of the intermediate scattering function is very similar to that in the SCNP bulk. This more retarded relaxation with respect to the linear chains would be related to the relaxation of the internal loops in the SCNPs as argued for the SCNPs in the bulk.^40,50^ We could also tentatively suggest the interpenetration of the precursor chains within the SCNPs as an explanation of the observed heterogeneity enhancement in the SCNPs’ dynamics. Using coarse-grained and atomistic simulations on PS-based nanocomposites, it was possible to demonstrate infiltration of matrix monomers into the interior of the SCNPs.^26^ This finding, supporting also experimental observations previously reported on PMMA/PEO nanocomposites,^21^ leads to a close correlation between SCNPs and matrix chains. Local entanglements produced by the linear chains on the SCNP strands could give rise to more heterogeneous dynamics of the “fillers” at local length scales and to an increase of the average length of the jumps involved in the sublinear diffusive regime of the atoms in the SCNPs.

**Figure 8:**
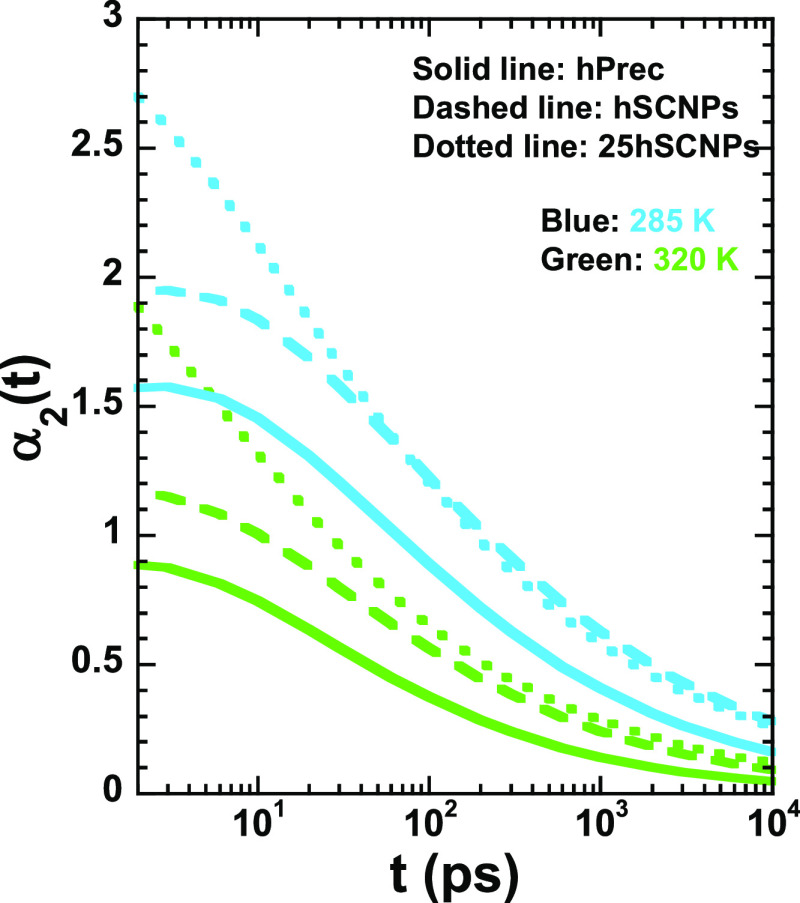
Non-Gaussian parameter obtained from the AJD description of the QENS results on the pure precursor melt (solid lines), the melt of SCNPs (dashed lines), and the SCNPs in the nanocomposite (dotted lines). Different colors correspond to 285 K (blue) and 320 K (green).

We may exploit the description in terms of the AJD model to provide a more quantitative estimation of the non-Gaussian effects induced, first, by intramolecular cross-linking (melt of SCNPs versus melt of precursor chains) and, later, on the SCNPs by the presence of the linear chains in the nanocomposite. In the framework of the AJD model, the time-dependent second-order non-Gaussian parameter α_2_(*t*) can be calculated as
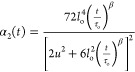
5The results obtained using the values of the parameters shown in Table 1 can be seen in Figure 8 for precursors, SCNPs and SCNPs in the nanocomposite, for two of the temperatures investigated. The values of this parameter become smaller with increasing temperature. For a given temperature, an increase is found from the melt of linear chains to the melt of SCNPs, as reported in ref (45) from MD simulations. When the SCNPs are embedded in the nanocomposite, an additional increase of α_2_(*t*) can be seen at short times. This could be associated with the fraction of segments in contact with the faster linear precursor at the interface. We note that non-Gaussian diffusion of SCNPs found in coarse-grained MD simulations has been attributed to the dynamic coupling at a specific length scale with the polymer melt chain segments.^51^

**Figure 9:**
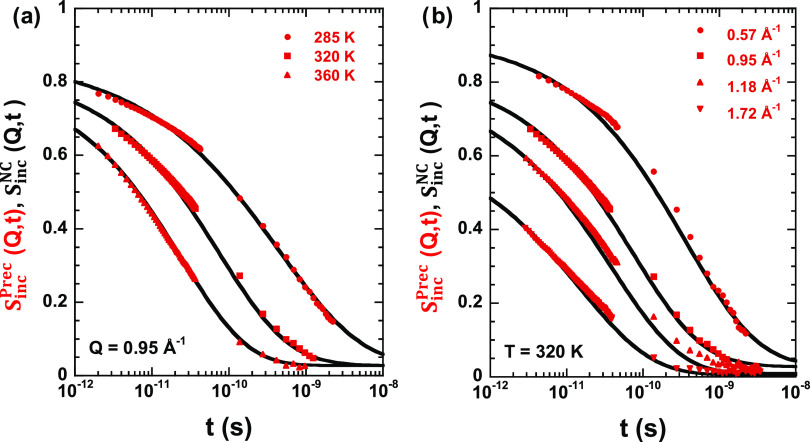
Intermediate scattering function calculated for all of the hydrogens in the nanocomposite (eq 6) (lines) starting from the KWW functions describing the component behavior (solid lines in Figure 4) compared with the experimentally determined intermediate scattering function of the precursor melt (symbols).^33^ Panel (a) shows the results for *Q* = 0.95 Å^–1^ at the different temperatures explored and panel (b) at 320 K for the different *Q*-values investigated.

We may ask now whether our microscopic results are compatible with the macroscopic observations by DSC. This technique is not sensitive to any of the particular components but reflects the averaged behavior in the sample. On the contrary, thanks to isotopic labeling we have been able to separately characterize by QENS the intermediate scattering function of both components in the nanocomposite *S*_inc_^Prec/NC^(*Q*,*t*) and *S*_inc_^SCNP/NC^(*Q*,*t*). Starting from this information, we can easily “construct” the total intermediate scattering function of all hydrogens in the system, the function that would have been measured on a fully protonated sample, *S*_inc_^NC^(*Q*,*t*)
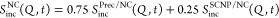
6This function gives the averaged response of the system. We have calculated eq 6 using the KWW fitting functions describing the experimental results on *S*_inc_^Prec/NC^(*Q*,*t*) and *S*_inc_^SCNP/NC^(*Q*,*t*) (lines in Figure 4). The results of this calculation are shown in Figure 9 as lines. For comparison, we have plotted in this figure also the intermediate scattering function measured on the pure precursor chains in the melt under the same conditions.^33^ As can be seen in this figure, the calculated function for the whole nanocomposite perfectly matches the results observed in the linear precursor melt. This is exactly the observation we have reported above from the DSC experiments. Thus, our QENS results on the microscopic dynamics well above the glass transition temperature are perfectly compatible with the calorimetric observation that the properties of the mixture appear to mimic completely the pure matrix behavior.

In the seminal work of Mackay et al.,^22^ both *T*_g_ reduction and viscosity reduction in PS-based nanocomposites were reported. In the mixtures investigated in that work, contrarily to our case, the *T*_g_ was the same for the two neat components. We could expect that, given that in our case the segmental mobility of the precursor is accelerated by the presence of the SCNPs, the *T*_g_ of the nanocomposite would have decreased if the *T*_g_ of the SCNPs remained the same as for the linear precursor chains. Thus, our results are perfectly compatible with those reported in ref (22) regarding the effects on the glass transition. We could expect that the interfacial speed up of the matrix segmental relaxation, noticed here thanks to component selectivity offered by QENS, in agreement with the MD simulation prediction of refs (25, 26), would be at the origin of the observed reduction of *T*_g_ in ref (22). We note in passing that in the case of our nanocomposites, no *T*_g_ reduction was observed either for a sample with lower concentrations of SCNPs (10%) (see Figure 1). On the other hand, our experiments do not address the impact of SCNPs on viscosity. Recent MD simulations have found an unexpected amplification of the viscosity reduction with an increasing molecular weight of the polymers in the matrix.^52^ This is a certainly very important issue that shall be the subject of future investigations.

## Conclusions

QENS techniques together with isotopic labeling have allowed disentangling the component dynamics in an all-polymer nanocomposite consisting of 25% SCNPs in a 75% polymer matrix composed of the linear precursor chains of the SCNPs. In the raw materials, the intramolecular cross-links induce a slowing down, additional deviations from Gaussian behavior, and a broadening of the dynamic response of the SCNPs with respect to the reference precursors. Dynamic asymmetry is also patent in the nanocomposite. We observe the development of dynamic heterogeneity in the intermediate scattering function of the NC components, which grows with increasing time: while at times shorter than ≈10 ps, the intermediate scattering functions of the precursor and SCNPs are similar, they become more and more distinct at longer times. The more retarded dynamics of the SCNPs with respect to the linear chains would be related to the relaxation of the internal loops in the SCNPs as argued for the SCNPs in the bulk. This is a mechanism that determines the final relaxation at long times. In the nanocomposite, the displacements of SCNPs’ hydrogens show enhanced deviations from Gaussian and exponential behavior compared with the pure melt of SCNPs. These effects would be due to the speed up of the motions of the SCNPs at short times induced by the surrounding faster linear precursor dynamics, particularly at the interface. On the other hand, the motions in the linear matrix are faster than in the bulk precursor material. Acceleration of the segmental relaxations of the melt chains was also reported by MD simulations^25^ and attributed to the thermal deformations of the loose SCNP surface structures. These combined effects result in an averaged behavior that coincides with that of the pure precursor. This is in accordance with the macroscopic observations by DSC experiments, from which no impact of the presence of SCNPs on the material with respect to the pure matrix dynamics is deduced. Our study thus demonstrates the power of QENS combined with isotopic labeling to selectively characterize component dynamics at the microscopic level in complex materials like all-polymer nanocomposites and resolve subtle effects that are overlooked by macroscopic nonselective methods.
